# Sex-specific element accumulation in honey bees (*Apis mellifera*)

**DOI:** 10.1007/s11356-024-32822-z

**Published:** 2024-03-13

**Authors:** Nenad M. Zarić, Robert Brodschneider, Walter Goessler

**Affiliations:** 1 Faculty of Biology, University of Belgrade, Studentski Trg 16, 11000 Belgrade, Serbia; 2 Analytical Chemistry for Health and Environment, Institute of Chemistry, University of Graz, Universitaetsplatz 1, 8010 Graz, Austria; 3 Institute of Biology, University of Graz, Universitaetsplatz 2, 8010 Graz, Austria

**Keywords:** Sexual dimorphism, Element composition, Drones, Workers, Food filtration, ICPMS

## Abstract

Honey bees are social insects that show division of labor and sexual dimorphism. Female honey bees differentiate in two different castes, queens or worker bees, while males are called drones. Worker bees have different tasks in the hive including collection of food, its processing, caring for brood, protecting the hive, or producing wax. The drones’ only role is to mate with a virgin queen. Many studies have dealt with differences in physiology, behavior, and morphology of workers and drones. This is the first study that demonstrates differences in element accumulation and composition between workers and drones honey bees. Using inductively coupled plasma mass spectrometry, we found that worker honey bees have higher concentrations of most elements analyzed. Drones had higher concentrations of elements essential to bees, Na, P, S, Zn, Cu, and especially Se (2.2 × higher), which is known to be important for sperm quality and fertility in many animals. Until now higher Se content was not observed in male insects. These differences can be attributed to different environmental exposure, reproductive role of drones, but mostly to the food workers and drones consume. Worker bees feed on bee bread, which is rich in minerals. Drones are fed food pre-processed by worker bees.

**Supplementary Information:**

The online version contains supplementary material available at 10.1007/s11356-024-32822-z.

## Introduction

Honey bees are social insects that show different sexes and castes. Female honey bees differentiate in two different castes, queens or worker bees. The queen, usually only one in the hive, is the reproductive female. Her role is to lay eggs and regulate the hive’s activity with pheromones (Pankiw et al. 1998). Queens develop from larvae that are only fed an exclusive nutrient rich food called “royal jelly,” produced in the hypopharyngeal and mandibular glands of worker bees (Mao et al. 2024). The second female castes are non-reproductive workers. They develop from fertilized eggs but are fed royal jelly only for the first three days and afterwards are fed worker jelly, a brood food containing honey and beebread (Mao et al. 2024). Worker jelly is nutrient diluted compared to royal jelly (Sagili et al. 2018; Wang et al. 2016). Queen larvae not only receive food of better quality but also the quantity of their food is greater (Slater et al. 2020). The third group is comprised of male bees called drones, originating from unfertilized eggs. Drones are fed royal jelly for the first three days, the same as female larvae. After that drone larvae are fed drone jelly, which contains significant amounts of pollen (Haydak 1957; Matsuka et al. 1973). Weight gain of drones is significantly higher compared to workers during the larval period (Hrassnigg and Crailsheim 2005). This is also observed at the emergence of young adults. Drones are typically 2x to 2.5 × heavier than workers (Bowen-Walker and Gunn 2001; Duay et al. 2003).

Workers, depending on their age, have different roles in the hive. They take care of the brood; build and protect the hive; forage for food and water; process food; nourish the workers, drones, and queens; and produce wax (Schmickl and Crailsheim 2004, 2002). To be able to perform all these tasks, workers are equipped with well-developed hypopharyngeal and mandibular glands, wax glands, and scent glands (Hrassnigg and Crailsheim 2005). On the other hand, the main role of drones is to produce sperm and mate with a virgin queen. The differences in worker and drone physiology are manyfold and include developmental time, nourishment, weights, body composition, energy metabolism, digestive physiology, behavior, or pathogen susceptibility (Brodschneider and Crailsheim 2010; Hrassnigg et al. 2005; Hrassnigg and Crailsheim 2005; Retschnig et al. 2014). The development of individual drone larvae costs nurse bees, and the whole colony, much more than the development of worker larvae (Haydak 1970). This can be seen by observing the weight of the larvae at the time of cell sealing. Worker larvae’s fresh weight is 144–162 mg, while drone larvae weigh 262–419 mg making them around 2x heavier (Hrassnigg and Crailsheim 2005). Most of the growth after emergence is contributed to the increase in protein content in both worker bees and drones (Haydak 1957). Pollen is the main source of protein for worker bees. Although drones do not consume pollen, they increase their protein content. This increase is associated to flight muscles and sexual organs (Hrassnigg and Crailsheim 2005). Drones that were isolated, without nursing workers, and were fed only pollen do not fully develop their mucus, which is associated to reproduction (Hrassnigg and Crailsheim 2005). This shows the importance of drones feeding of jelly by nurse bees for their development (Hrassnigg and Crailsheim 2005).

Workers and drones have different feeding. Workers ingest more pollen. It is used in their hypopharyngeal glands to produce proteinaceous secretions, which is then fed to the brood, queen, other workers, and drones (Crailsheim 1992). Drones eat much less pollen, only 2–3% compared to workers (Hrassnigg and Crailsheim 2005). They also have a smaller stomach capacity compared to workers (Snodgrass 1956). Most of the food drones consume is jelly fed to them by worker honey bees (Crailsheim 1992).

Worker bees, mostly foragers, are the ones that gather food for the entire colony. They fly out of the hive 12–15 times per day to gather food and water for the hive (Perugini et al. 2011). However, the authors have observed that worker bees as young as 6 days can gather pollen. Through their flight, bees can be exposed to the outside environment and different elements present in it. On the other hand, drones mostly stay in the hive and do only short orientation and defecation flights. They fly to drone congregation sites just for a short period of the day only if the weather is suitable (Szolderits and Crailsheim 1993). In this way, they are not exposed to the hives outside the environment as much as worker honey bees, especially foragers.

Honey bee body composition of macromolecules is well studied (Helm et al. 2017; Kunert and Crailsheim 1988), but elemental composition, including metals, is less investigated. The origin of metals, metalloids, and non-metals in the environment can be natural or anthropogenic (Yu et al. 2023). They are non-degradable, meaning that they can only change their chemical form and enter biological systems (Perugini et al. 2011). Some metals are essential parts as enzyme co-factors. These include Cu, Mo, Co, and Cr (Gordon 1959). Zn and Mn are essential for hardening of insect mandibles cuticle (Behmer 2008). A central element in cytochrome enzymes is Fe (Behmer 2008). It was proven that if some pollen species are deficient in Na, K, S, P, N, Cu, and Zn, it could hinder bee growth and development, specifically in larval stages, which influences their adult traits such as size, fertility, immunity, and lifespan (Filipiak et al. 2017). For essential elements, it is known that lower doses can be very beneficial, but higher doses are toxic to honey bees. This is also true for Se, where this line of beneficial to toxic dose is very narrow (Alburaki et al. 2019; Burden et al. 2016). Some elements, such as Al, Ba, Cd, Ni, Pb, and Sr, are considered non-essential and might interact with macromolecules by replacing essential metals and therefore could potentially be toxic (AL Naggar et al. 2020; Chicas-Mosier et al. 2017; Farias et al. 2023; Monchanin et al. 2021; Schmarsow et al. 2023).

Elemental analysis in honey bees has been the subject of many studies. Most of these studies used bees to monitor element pollution in the environment (Barbosa et al. 2021; Conti et al. 2022; Farias et al. 2023; Fry et al. 2023; Hladun et al. 2016; Smith and Weis 2022; van der Steen et al. 2016; Zarić et al. 2018a, b; Zarić et al. 2022; Zhou et al. 2018). Some focused on essential and non-essential elements, not just in whole honey bees, but in their hemolymph as well (Ilijević et al. 2021). Usually, these studies are done on pooled homogenized bee samples. Only one previous study was done on 31 element composition of 337 individual bees (Zarić et al. 2021).

Although there are studies that report element deposition in drones and workers (Ćirić et al. 2021; Filipiak et al. 2017), these two groups were never directly compared for the differences in their elemental composition. These differences were generally not studied in adult insects of different sexes. The aim of this study is to determine the differences in element composition between male (drone) and female (worker) honey bees. For this study, individual workers and drones were analyzed for their content of 31 different elements.

## Materials and methods

### Sample collection

Honey bee samples were taken at two different time points and two different apiaries in Graz. At each apiary worker and drones were taken from the same hive. One apiary was located in Gries, Graz, Austria. The apiary was in the city center on a building roof. Samples in Gries were collected in June 2021. Second apiary was located at the University of Graz in the city center, approximately 3 km from the apiary in Gries. Samples at University of Graz were taken in August 2023. Samples of adult worker honey bees (*n* = 27) were collected from the outer most frame that had honey on it but no brood, as it is believed that these are mostly forager bees that have already flown out of the hive (Bilalov et al. 2015; Van der Steen et al. 2012). Drones were collected throughout the hive (*n* = 21). Individual bees (worker or drones) were placed into separate Eppendorf 2 mL tubes. After collection they were frozen at − 80 °C and kept in the freezer until analyses.

### Chemicals and standards

Purification system (Milli-Q, Merck Millipore, Darmstadt, Germany) was used to provide purified water (18.2 MΩ cm). Nitric acid (HNO_3_) Rotipuran p. a. ≥ 65% (Carl Roth, Karlsruhe, Germany) was subboiled with a MLS duoPUR (MLS, Leutkirch, Germany) prior to its use for the preparation of samples. For internal standards and preparation of calibration standards, we used ICP Single-Element Standards Certipur (Merck Millipore, Darmstadt, Germany) and Single Element Standards for ICP (Carl Roth, Karlsruhe, Germany). Fifteen and fifty mL Cellstar polypropylene tubes (Greiner Bio-One International GmbH, Kremsmünster, Austria) were used for preparation of all solutions.

### Sample preparation

The samples were freeze-dried before analyses. Afterwards, individual worker or drone honey bees were weight into clean 10 mL quartz vessels. The digestion was done using an ultraCLAVE IV microwave digestion system (MLS GmbH, Leutkirch, Germany) with 2 mL conc. HNO_3_ and 3 mL ultrapure water. With each digestion, three digestion blanks (2 mL conc. HNO_3_ and 3 mL ultrapure water) and three reference materials 8414 “Bovine muscle powder” (NRC, Canada) (~ 0.25 g and 5 mL conc. HNO3) were analyzed. After the loading pressure of 40 bars inside the vessels was achieved by high purity Argon 5.0, the microwave heating program was started. The temperature was raised gradually to 80 °C in 10 min, ramped to 150 °C in further 25 min, then ramped to 250 °C in 20 min and finally held at 250 °C for 30 min. After cooling, the digestion solutions were transferred to 50 mL Cellstar tubes and diluted with ultrapure water to a final volume for blanks and samples of 20 mL and reference material 50 mL (10% (*v/v*) nitric acid).

### Determination of element concentrations

All element concentrations were determined using inductively coupled plasma mass spectrometry–ICPMS (Agilent ICPMS 7700x, Waldbronn, Germany). For 32 elements, an external calibration curve in four different concentration ranges and with six points each was made in 10% HNO_3_ (0.0100–5.00 μg L^−1^ for Li, V, Cr, Co, Ni, As, Se, Mo, Ag, Cd, Sn, Sb, Cs, Tl, Pb, and U; 0.1–50 μg L^−1^ for B, Ba, Cu, Rb, and Sr; 1.00–500 μg L^−1^ for Al, Mn, Fe, and Zn; 100–50,000 μg L^−1^ for Na, K, Ca, Mg, P, and S). Instrument performance is reported in Table S1. Selected mass, tune mode, and internal standard for correction for each element analyzed are reported in Table S2.

### Quality control

Internal standard solution containing 200 μg L^−1^ of Be, Ge, In, and Lu in a matrix of 1% v/v HNO_3_ was continuously added for instrument stability control. In addition, drift standards were measured every ten samples. The accuracy was evaluated using two reference materials: SRM 1640a Trace elements in natural water (National Institute of Standards & Technology, Gaithersburg, USA) and CRM 8414 Bovine muscle powder (NRC, Canada) (Supplementary material, Table S3 and S4).

### Statistical analyses

For statistical analysis, Microsoft Excel 2021, IBM SPSS Statistics 27, and PAST 4.03 were used. To assess statistically significant differences between female (worker) and male (drones) honey bees, we used perMANOVA (PAST 4.03) and MANOVA (SPSS 27). To determine statistically significant differences between individual elements, both parametric MANOVA (tests of between-subjects effects) and Kruskal–Wallis *H* test were applied to the dataset (SPSS 27). In MANOVA, Wilk’s *Λ* is a measure of the percent variance in dependent variables not explained by differences in levels of the independent variable, and partial *η*^2^ gives information on how large of an effect the independent variables had on the dependent variable. For NDMS ordinary plot, Bray–Curtis distance was used (PAST 4.03).

## Results and discussion

Average dry weight of collected worker honey bees was 42 ± 14 mg, which is higher than in our previous study (29.2 ± 5.8 mg) for bees from Serbia (Zarić et al. 2021), or the one done by (Brodschneider et al. 2009) in Graz. Drones’ average dry weight was 63.7 ± 4.4 mg, which is a bit higher than reported in the literature (from 30.7 to 56.9 mg (according to Henderson 1992)). In this study, drones have approximately 30% higher dry weight compared to worker bees. There were no literature data for dry weight comparison; however, fresh weight drones are usually twice heavier compared to worker bees (Es’kov and Es’kova 2013; Hrassnigg and Crailsheim 2005).

**Fig. 1:**
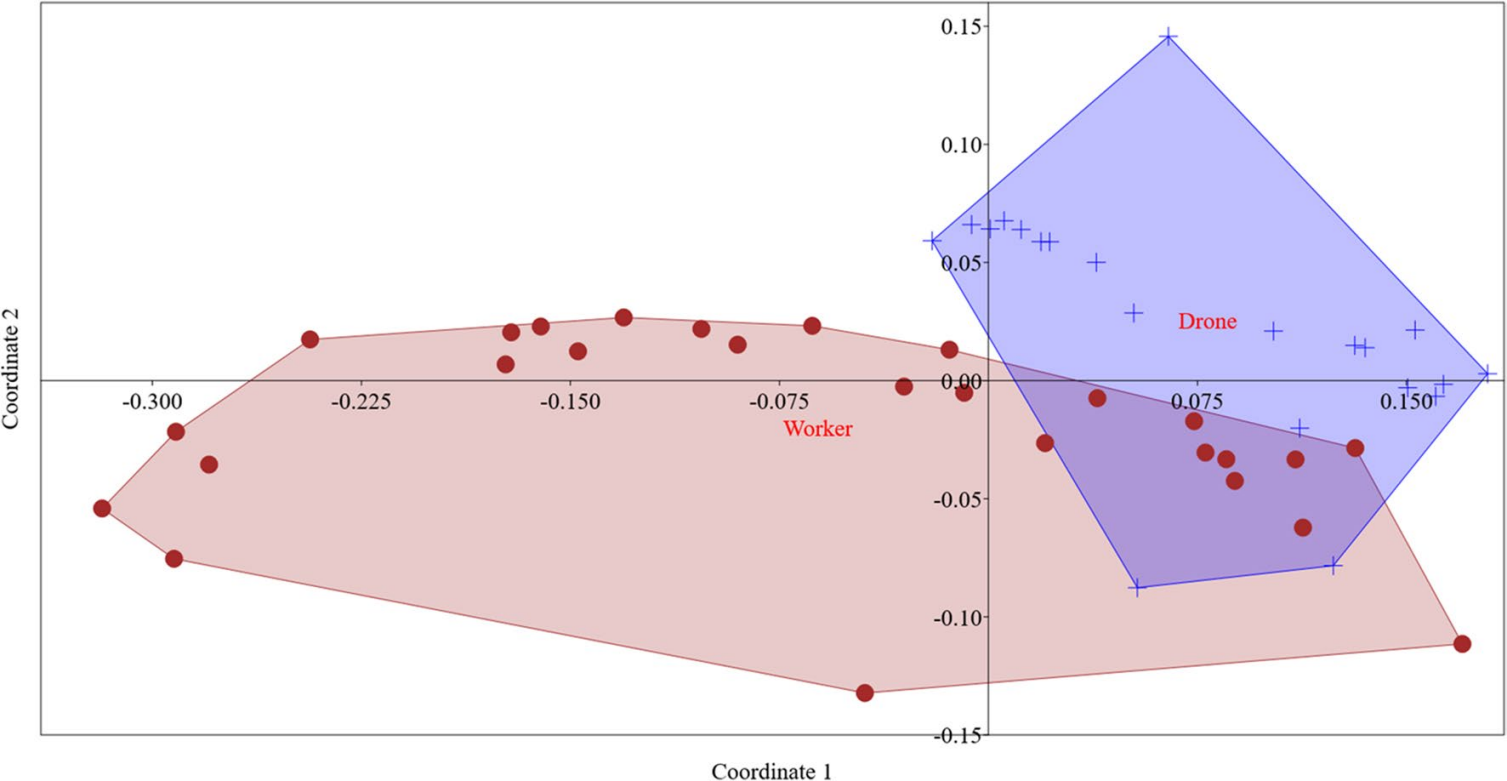
NMDS ordination plot; red circles represent workers; blue + represent drones (MANOVA, *p* < 0.001)

Out of the 32 analyzed elements, 27 were above the detection limit (LOD). Elements below LOD (Li, Cs, Hg, Tl, and U) were discarded from further discussion. The three most abundant elements in both workers and drones are K > P > S, while the lowest concentrations were observed for Sb > Ag (Table 1). PerMANOVA (*F* = 14.55, *p* < 0.005) and MANOVA (*F* (27, 20) = 62.02, *p* < 0.0005; Wilk’s *Λ* = 0.12, partial *η*^2^ = 0.99) showed that there was a statistically significant difference between drones and workers. Two distinctive groups can be seen in non-metric multidimensional scaling (NMDS) ordination plot (Fig. 1), confirming that there are differences in element profile between workers and drones.

**Table 1:** Concentration of elements (mg kg^−1^ dry weight ± standard deviation) in worker (*n* = 27) and drone (*n* = 21) honey bees

Element	Workers	Drones
Ag**	0.013 ± 0.010	0.0059 ± 0.0016
Al**	16.0 ± 7.8	8.4 ± 5.2
As**	0.057 ± 0.029	0.033 ± 0.013
B	6.0 ± 2.7	5.3 ± 1.6
Ba	1.64 ± 0.61	1.5 ± 1.1
Ca**	953 ± 244	717 ± 191
Cd**	0.094 ± 0.059	0.0165 ± 0.0077
Co**	0.14 ± 0.13	0.053 ± 0.031
Cr	0.12 ± 0.11	0.060 ± 0.038
Cu**	21.7 ± 5.4	26.2 ± 3.1
Fe**	169 ± 60	107 ± 17
K	9433 ± 2157	10456 ± 1636
Mg	900 ± 274	991 ± 129
Mn*	82 ± 56	43 ± 40
Mo**	0.75 ± 0.22	0.304 ± 0.053
Na**	457 ± 113	939 ± 249
Ni**	0.33 ± 0.21	0.124 ± 0.050
P**	6629 ± 1991	8755 ± 1230
Pb**	0.160 ± 0.060	0.107 ± 0.043
Rb**	10.6 ± 3.2	6.3 ± 2.8
S**	3728 ± 1075	5617 ± 649
Sb**	0.025 ± 0.015	0.0083 ± 0.0041
Se**	0.191 ± 0.073	0.43 ± 0.12
Sn	0.032 ± 0.017	0.026 ± 0.014
Sr	1.30 ± 0.36	1.13 ± 0.69
V**	0.035 ± 0.015	0.0127 ± 0.0065
Zn*	94 ± 32	115 ± 19

MANOVA: *p < 0.05 and **p < 0.01

Most of the analyzed elements had statistically significant differences between workers and drones in both parametric and non-parametric tests (Supplementary material, Table S5). There were seven elements that did not show statistically significant differences (B, Ba, Cr, K, Mg, Sn, and Sr). All of them except K had slightly higher concentrations in workers (Table 1).

### Workers

Significantly higher concentrations in worker bees compared to drones were observed for Al, Ca, V, Mn, Fe, Co, Ni, As, Rb, Mo, Ag, Cd, Sb, and Pb (Table 1). The worker honey bees that were sampled should be mostly foragers according to Bilalov et al. (2015). However, it could be that the sampled bees contain both foragers and house bees. Foragers are the bees that fly out 12–15 times per day to gather food and water for the hive (Perugini et al. 2011). These bees have been exposed to the full impact of the environment and the pollution present in water, soil (through plant pollen and nectar), and air (Hladun et al. 2015; Sadeghi et al. 2012; Zarić et al. 2017). Worker bees consume bee bread as a protein source, the pollen harvested by pollen foragers, deposited in cells and ripened there for a few days (Roessink and van der Steen 2021). Hence, although it cannot be claimed that most of the sampled bees are foragers, we know that all of sampled bees consumed pollen, either directly or from bee bread.

### Drones

Higher concentration in drones compared to foragers can be observed for Na, P, S, Cu, Zn, and Se. Out of these for insects Na, P, S, Cu, and Zn are considered essential (Filipiak et al. 2017; Nation 2015). Cu is an important part of enzymes (Gordon 1959). For most of these elements, further investigation is needed to explain their higher concentrations in drones. Out of all the elements that had higher concentrations in drones, Se was the one with the biggest difference. It was more than twofold higher in drones compared to foragers. It was proven that Se is very important for fertility and sperm quality in many animals and man (Alavi et al. 2020; Hansen and Deguchi 1996; Xu et al. 2022). For insects, Se could be beneficial for egg fertilization (Martin-Romero et al. 2001). Considering that the drones’ main role is to produce sperm and mate with a queen, we assume that higher Se content is due to an active accumulation in their sperm, which is worth further study.

Lower concentrations of most other, especially non-essential, elements in drones compared to worker bees are likely due to their lifecycle. Drones fly out during mating season only once per day and only if the weather conditions are optimal (Hrassnigg and Crailsheim 2005). If they do not mate within 30 min, they return to the hive. In comparison to foragers that spend most of the day outside of the hive gathering food, drones are most of the day inside. They are not as much exposed to the outside environment. As already mentioned, diet can also have an influence on element concentrations. In contrast to worker bees, drones never consume bee bread but are fed processed protein jelly by nurse bees (Crailsheim 1992; Hrassnigg and Crailsheim 2005). In a recent study by Taylor et al. (2023), it was concluded that elements are not attached to the surface of the bee, but are bioaccumulated in the honey bee body. This was confirmed by our own experiments on washed bees (unpublished data). Most of the elements honey bees accumulate are from the food they eat (Gekière et al. 2023). While worker bees eat unprocessed food, drones are fed nectar or honey and protein jelly. Drones are missing hypopharyngeal glands (glands that produce food), wax glands, and most of the structures to collect food (Hrassnigg and Crailsheim 2005). They also have a slenderer honey stomach compared to workers. Drones consume only 2–3% of pollen that worker bees do (Szolderits and Crailsheim 1993).

The finding that non-essential elements have lower concentrations in drones supports that worker bees filtrate food and hence do not pass on non-essential or non-beneficial materials in the processed food, as demonstrated by Lucchetti et al. (2018) for larval feeding (Végh et al. 2021). Most of the food that drones get is pre-digested, via proteinaceous glandular secretions and honey provided by workers. A study done on Pb concluded that most of it is located in the midgut and is not passed on to the food they produce (Raes et al. 1992). It could be that worker honey bees, especially nurse bees, have a mechanism for filtering unwanted elements from food, in this case pollen.

## Conclusions

This work shows that there are differences in element accumulation between the sexes of honey bees. Significand differences were observed for 24 out of 27 detected elements. Drones had higher concentration only for essential elements, Na, P, S, Zn, Cu, and Se. The rest of the elements had significantly higher concentrations in worker bees. Se is known to be important for sperm quality and fertility in many animals and humans. This is the first time it was observed that male insects have higher Se content compared to females. For the rest of the elements, a couple of factors could influence these differences. Sampled bees are most likely a mixture of worker bees and foragers that spend most of their time outside of the hive gathering food. Hence, they are more exposed to environmental pollution, compared to drones that spend most of their life inside the hive. However, most likely, explanation is in the food they consume. Worker honey bees feed on unprocessed food from the environment, mostly bee bread, which is rich in minerals. Drones on the other hand are fed pre-digested, “filtered” food produced by worker bees. The underlying mechanism of filtering non-essential elements in honey bees is still unknown and needs further study.

## Supplementary Information

Below is the link to the electronic supplementary material.

Supplementary file1 (DOCX 39 KB)

## Data Availability

Original data is provided in the supplementary material.
