# Correction to: Sex-specific element accumulation in honey bees (*Apis mellifera*)

**DOI:** 10.1007/s11356-024-32957-z

**Published:** 2024-03-18

**Authors:** Nenad M. Zarić, Robert Brodschneider, Walter Goessler

**Affiliations:** 1 Faculty of Biology, University of Belgrade, Studentski Trg 16, 11000 Belgrade, Serbia; 2 Analytical Chemistry for Health and Environment, Institute of Chemistry, University of Graz, Universitaetsplatz 1, 8010 Graz, Austria; 3 Institute of Biology, University of Graz, Universitaetsplatz 2, 8010 Graz, Austria

**Correction to: Environmental Science and Pollution Research**

10.1007/s11356-024-32822-z

Robert Brodschneider was incorrectly affiliated to affiliation 2 and Walter Goessler was incorrectly affiliated to affiliation 3. The correct affiliation of Robert Brodschneider is affiliation 3 and Walter Goessler affiliation 2.

The Original article has been corrected.

