# Supplementary material for: Sex-specific element accumulation in honey bees (Apis mellifera)
Source: Environ Sci Pollut Res Int. 2024 Mar 13;32(16):10348–55. doi: 10.1007/s11356-024-32822-z (PMC11996938; doi:10.1007/s11356-024-32822-z)
Supplement: Supplementary file 1 — Supplementary file1 (DOCX 39 KB) [file 11356_2024_32822_MOESM1_ESM.docx]

Table S1. Performance of the ICPMS

| **Parameter** | **No-gas mode** | **Collision mode** | **Reaction mode** |
| --- | --- | --- | --- |
| Cell gas | - | He | H_2_ |
| ^7^Li [CPS per µg L^-1­^] | 14*10^3^ | - | - |
| ^59^Co [CPS per µg L^-1^] | - | 4.5*10^3^ | 2.6*10^3^ |
| ^89^Y [CPS per µg L^-1^] | 17*10^3^ | 3.4*10^3^ | 15*10^3^ |
| ^205^Tl [CPS per µg L^-1^] | 13*10^3^ | 7.9*10^4^ | 12*10^4^ |
| average RSD [%] | 2.3 | 2.9 | 2.7 |
| ^140^Ce^16^O/^140^Ce [%] | 1.3 | 0.5 | 1.3 |
| ^140^Ce^2+^/^140^Ce^+^ [%] | 1.3 | 2.9 | 1.1 |

Table S2. Selected mass, tune mode, internal standard and detection limits (LoD)

| **Monitored isotope** | **Tune mode** | **Internal standard** | **Detection limit^*^**  **(µg L^-1^)** |
| --- | --- | --- | --- |
| ^7^Li | No-gas | ^9^Be | 0.025 |
| ^11^B | No-gas | ^9^Be | 0.025 |
| ^23^Na | He | ^9^Be | 27 |
| ^24^Mg | He | ^9^Be | 11 |
| ^27^Al | No-gas | ^9^Be | 2.4 |
| ^31^P | He | ^9^Be | 177 |
| ^32^S | He | ^9^Be | 302 |
| ^39^K | He | ^9^Be | 9.8 |
| ^43^Ca | He | ^9^Be | 257 |
| ^51^V | He | ^74^Ge | 0.02 |
| ^53^Cr | He | ^74^Ge | 0.08 |
| ^55^Mn | He | ^74^Ge | 0.55 |
| ^56^Fe | He | ^74^Ge | 1.4 |
| ^59^Co | He | ^74^Ge | 0.04 |
| ^60^Ni | He | ^74^Ge | 0.9 |
| ^65^Cu | He | ^74^Ge | 0.24 |
| ^66^Zn | He | ^74^Ge | 18 |
| ^75^As | He | ^74^Ge | 0.0030 |
| ^78^Se | H_2_ | ^74^Ge | 0.01 |
| ^85^Rb | He | ^74^Ge | 0.023 |
| ^88^Sr | He | ^74^Ge | 0.1 |
| ^98^Mo | No-gas | ^74^Ge | 0.02 |
| ^107^Ag | No-gas | ^115^In | 0.001 |
| ^111^Cd | No-gas | ^115^In | 0.009 |
| ^118^Sn | No-gas | ^115^In | 0.02 |
| ^121^Sb | No-gas | ^115^In | 0.01 |
| ^133^Cs | No-gas | ^115^In | 0.002 |
| ^137^Ba | No-gas | ^115^In | 0.08 |
| ^205^Tl | No-gas | ^175^Lu | 0.0012 |
| ^208^Pb | No-gas | ^175^Lu | 0.03 |
| ^238^U | No-gas | ^175^Lu | 0.0003 |

^*^LoD = mean_blanks_ + 3*σ_blanks_

Table S3. Certified and determined values for elements in NIST SRM 1640a Trace Elements in Natural Water

| **Element** | **Cert. mass conc. [µg L^-1^]** | | | **Analyzed mass conc. [µg L^-1­^] (n=4)** | | |
| --- | --- | --- | --- | --- | --- | --- |
| Li | 0.4034 | ± | 0.0094 | 0.414 | ± | 0.029 |
| B | 300.7 | ± | 3.1 | 276.6 | ± | 4.6 |
| Na | 3137 | ± | 31 | 2985 | ± | 299 |
| Mg | 1058.6 | ± | 4.1 | 1018 | ± | 106 |
| Al | 53.0 | ± | 1.8 | 50.6 | ± | 3.7 |
| K | 579.9 | ± | 2.3 | 591 | ± | 78 |
| Ca | 5615 | ± | 21 | 5816 | ± | 725 |
| V | 15.05 | ± | 0.25 | 13.97 | ± | 0.82 |
| Cr | 40.54 | ± | 0.30 | 39.4 | ± | 1.7 |
| Mn | 40.39 | ± | 0.36 | 38.2 | ± | 1.7 |
| Fe | 36.8 | ± | 1.8 | 37.7 | ± | 3.2 |
| Co | 20.24 | ± | 0.24 | 18.85 | ± | 0.64 |
| Ni | 25.12 | ± | 0.14 | 23.27 | ± | 0.52 |
| Cu | 85.75 | ± | 0.51 | 85.20 | ± | 0.68 |
| Zn | 55.64 | ± | 0.35 | 52.1 | ± | 1.9 |
| As | 8.075 | ± | 0.070 | 7.79 | ± | 0.12 |
| Se | 20.13 | ± | 0.17 | 18.45 | ± | 0.97 |
| Rb | 1.198 | ± | 0.011 | 1.19 | ± | 0.14 |
| Sr | 126.03 | ± | 0.91 | 114.9 | ± | 8.5 |
| Mo | 45.60 | ± | 0.61 | 43.2 | ± | 2.1 |
| Ag | 8.081 | ± | 0.046 | 9.23 | ± | 0.30 |
| Cd | 3.992 | ± | 0.074 | 3.70 | ± | 0.18 |
| Sb | 5.105 | ± | 0.046 | 4.733 | ± | 0.082 |
| Ba | 151.80 | ± | 0.83 | 140.0 | ± | 6.9 |
| Tl | 1.619 | ± | 0.016 | 1.439 | ± | 0.073 |
| Pb | 12.10 | ± | 0.05 | 11.20 | ± | 0.27 |
| U | 25.35 | ± | 0.27 | 24.21 | ± | 0.38 |

Table S4 Certified and determined values for elements in CRM BOVN-1 Bovine Muscle Powder (*information values)

| **Element** | **Cert. mass conc. [mg kg^-1^]** | | | **Analyzed mass conc. [mg kg^-1­^] (n=12)** | | |
| --- | --- | --- | --- | --- | --- | --- |
| Na | 2100 | ± | 100 | 1799 | ± | 99 |
| Mg | 960 | ± | 95 | 842 | ± | 51 |
| Al* | 1.7 | ± | / | 1.14 | ± | 0.40 |
| P | 8360 | ± | 450 | 6911 | ± | 497 |
| S* | 8000 | ± | / | 6847 | ± | 266 |
| K | 15200 | ± | 400 | 13047 | ± | 884 |
| Ca | 145 | ± | 20 | 122 | ± | 10 |
| V* | 0.005 | ± | / | 0.00200 | ± | 0.00087 |
| Cr* | 0.071 | ± | / | 0.056 | ± | 0.011 |
| Mn | 0.37 | ± | 0.09 | 0.290 | ± | 0.021 |
| Fe | 71.2 | ± | 9.2 | 68.7 | ± | 2.8 |
| Co | 0.007 | ± | 0.003 | 0.00513 | ± | 0.00022 |
| Ni* | 0.05 | ± | / | 0.238 | ± | 0.017 |
| Cu | 2.84 | ± | 0.45 | 2.32 | ± | 0.39 |
| Zn | 142 | ± | 14 | 128.8 | ± | 5.0 |
| As | 0.009 | ± | 0.003 | 0.00732 | ± | 0.00044 |
| Se | 0.076 | ± | 0.010 | 0.0626 | ± | 0.0023 |
| Rb | 28.7 | ± | 3.5 | 24.7 | ± | 1.5 |
| Sr | 0.052 | ± | 0.015 | 0.0547 | ± | 0.0090 |
| Mo | 0.08 | ± | 0.06 | 0.0593 | ± | 0.0028 |
| Cd | 0.013 | ± | 0.011 | 0.0151 | ± | 0.0031 |
| Cs* | 0.05 | ± | / | 0.035 | ± | 0.013 |
| Ba* | 0.05 | ± | / | 0.0228 | ± | 0.0034 |
| Pb | 0.38 | ± | 0.024 | 0.32 | ± | 0.10 |

Table S5. Average concentrations of analyzed elements in worker and drones from two apiaries and two sampling years

|  | Gries, 2021 | | Uni-Graz, 2023 | |
| --- | --- | --- | --- | --- |
| Element | Worker | Drone | Worker | Drone |
| Ag | 0.020 ± 0.011 | 0.0057 ± 0.0016 | 0.0043 ± 0.0021 | 0.0043 ± 0.0021 |
| Al | 17.4 ± 7.6 | 10.2 ± 4.7 | 11.8 ± 5.0 | 4.5 ± 3.0 |
| As | 0.0700 ± 0.029 | 0.037 ± 0.017 | 0.0323 ± 0.0077 | 0.0285 ± 0.0048 |
| B | 4.6 ± 1.2 | 4.4 ± 1.4 | 8.9 ± 4.3 | 6.3 ± 1.1 |
| Ba | 1.55 ± 0.57 | 0.68 ± 0.32 | 2.0 ± 1.0 | 2.68 ± 0.46 |
| Ca | 872 ± 280 | 582 ± 119 | 1188 ± 276 | 898 ± 86 |
| Cd | 0.062 ± 0.035 | 0.0126 ± 0.0079 | 0.153 ± 0.045 | 0.0214 ± 0.0060 |
| Co | 0.066 ± 0.028 | 0.032 ± 0.015 | 0.33 ± 0.16 | 0.082 ± 0.022 |
| Cr | 0.16 ± 0.13 | 0.099 ± 0.062 | 0.0674 ± 0.0060 | 0.0311 ± 0.0023 |
| Cu | 21.6 ± 6.4 | 26.1 ± 2.8 | 24.1 ± 3.5 | 26.4 ± 3.7 |
| Fe | 145 ± 47 | 95 ± 21 | 209 ± 59 | 122 ± 11 |
| K | 8310 ± 1509 | 9557 ± 1587 | 12332 ± 1300 | 11781 ± 563 |
| Mg | 755 ± 192 | 892 ± 81 | 1104 ± 169 | 1127 ± 37 |
| Mn | 59 ± 36 | 12.2 ± 5.0 | 125 ± 65 | 96 ± 20 |
| Mo | 0.79 ± 0.25 | 0.273 ± 0.048 | 0.69 ± .013 | 0.334 ± 0.054 |
| Na | 423 ± 109 | 813 ± 176 | 513 ± 100 | 1152 ± 94 |
| Ni | 0.30 ± 0.21 | 0.149 ± 0.048 | 0.326 ± 0.084 | 0.090 ± 0.029 |
| P | 5494 ± 1395 | 7888 ± 687 | 8885 ± 639 | 10089 ± 502 |
| Pb | 0.183 ± 0.076 | 0.081 ± 0.035 | 0.140 ± 0.042 | 0.142 ± 0.021 |
| Rb | 9.2 ± 2.6 | 6.2 ± 3.8 | 13.2 ± 2.9 | 6.49 ± 0.47 |
| S | 3168 ± 832 | 5183 ± 378 | 4880 ± 598 | 6149 ± 493 |
| Sb | 0.030 ± 0.017 | 0.0107 ± 0.0038 | 0.0144 ± 0.0049 | 0.0051 ± 0.0013 |
| Se | 0.233 ± 0.052 | 0.518 ± 0.071 | 0.120 ± 0.039 | 0.310 ± 0.029 |
| Sn | 0.036 ± 0.018 | 0.0342 ± 0.0097 | 0.0226 ± 0.0094 | 0.016 ± 0.011 |
| Sr | 1.26 ± 0.41 | 0.62 ± 0.32 | 1.26 ± 0.37 | 1.96 ± 0.27 |
| V | 0.042 ± 0.021 | 0.0181 ± 0.0077 | 0.031 ± 0.014 | 0.0079 ± 0.0026 |
| Zn | 80 ± 30 | 107 ± 18 | 118 ± 21 | 129 ± 11 |

Table S5. Results of MANOVA and Kruskal-Wallis H test

| Element | MANOVA* | Kruskal-Wallis H test |
| --- | --- | --- |
| B | F(1,46)=2.66, p=0.109, partial η^2^=0.055 | H(2) = 0.327, p = 0.568 |
| Na | F(1,46)=80.3, p<0.0001, partial η^2^=0.636 | H(2) = 27.76, p < 0.0001 |
| Mg | F(1,46)=1.96, p=0.168, partial η^2^=0.041 | H(2) = 1.53, p = 0.216 |
| Al | F(1,46)=13.15, p<0.001, partial η^2^=0.222 | H(2) = 13.46, p < 0.0001 |
| P | F(1,46)=18.41, p<0.0001, partial η^2^=0.286 | H(2) = 12.41, p < 0.0001 |
| S | F(1,46)=40.55, p<0.0001, partial η^2^=0.469 | H(2) = 27.10, p <0.0001 |
| K | F(1,46)=2.51, p=0.120, partial η^2^=0.052 | H(2) = 3.16, p = 0.076 |
| Ca | F(1,46)=10.36, p<0.005, partial η^2^=0.184 | H(2) = 9.27, p = 0.002 |
| V | F(1,46)=26.38, p<0.0001, partial η^2^=0.364 | H(2) = 20.62, p < 0.0001 |
| Cr | F(1,46)=2.20, p=0.147, partial η^2^=0.045 | H(2) = 7.24, p = 0.007 |
| Mn | F(1,46)=6.34, p<0.05, partial η^2^=.0121 | H(2) = 7.36, p = 0.007 |
| Fe | F(1,46)=20.87, p<0.0001, partial η^2^=0.312 | H(2) = 16.34, p < 0.0001 |
| Co | F(1,46)=8.00, p<0.01, partial η^2^=0.148 | H(2) = 9.27, p = 0.002 |
| Ni | F(1,46)=16.21, p<0.0005, partial η^2^=0.261 | H(2) = 22.55, p < 0.0001 |
| Cu | F(1,46)=8.31, p<0.01, partial η^2^=0.153 | H(2) = 6.91, p = 0.009 |
| Zn | F(1,46)=6.33, p<0.05, partial η^2^=0.121 | H(2) = 5.37, p = 0.020 |
| As | F(1,46)=11.92, p<0.005, partial η^2^=0.206 | H(2) = 10.58, p = 0.001 |
| Se | F(1,46)=72.8, p<0.0001, partial η^2^=0.613 | H(2) = 31.84, p < 0.0001 |
| Rb | F(1,46)=20.49, p<0.0001, partial η^2^=0.308 | H(2) = 18.96, p < 0.0001 |
| Sr | F(1,46)=0.545, p=0.464, partial η^2^=0.012 | H(2) = 0.429, p = 0.513 |
| Mo | F(1,46)=86.30, p<0.0001, partial η^2^=0.652 | H(2) = 33.50, p < 0.0001 |
| Ag | F(1,46)=11.56, p<0.005, partial η^2^=0.201 | H(2) = 6.64, p = 0.010 |
| Cd | F(1,46)=23.19, p<0.0001, partial η^2^=0.335 | H(2) = 30.91, p < 0.0001 |
| Sn | F(1,46)=0.654, p=0.423, partial η^2^=0.014 | H(2) = 0.780, p = 0.377 |
| Sb | F(1,46)=20.51, p<0.0001, partial η^2^=0.308 | H(2) = 22.16, p < 0.0001 |
| Ba | F(1,46)=0.332, p=0.568, partial η^2^=0.007 | H(2) = 0.975, p = 0.324 |
| Pb | F(1,46)=12.47, p<0.005, partial η^2^=0.213 | H(2) = 9.65, p = 0.002 |

*Tests of between-subjects effects
